# Disrupting ceramide-CD300f interaction prevents septic peritonitis by stimulating neutrophil recruitment

**DOI:** 10.1038/s41598-017-04647-z

**Published:** 2017-06-27

**Authors:** Kumi Izawa, Akie Maehara, Masamichi Isobe, Yuka Yasuda, Makoto Urai, Yasutaka Hoshino, Keigo Ueno, Toshihiro Matsukawa, Mariko Takahashi, Ayako Kaitani, Emiko Shiba, Ayako Takamori, Shino Uchida, Koichiro Uchida, Keiko Maeda, Nobuhiro Nakano, Yoshinori Yamanishi, Toshihiko Oki, David Voehringer, Axel Roers, Susumu Nakae, Junko Ishikawa, Yuki Kinjo, Toshiaki Shimizu, Hideoki Ogawa, Ko Okumura, Toshio Kitamura, Jiro Kitaura

**Affiliations:** 1 Atopy Research Center, Juntendo University Graduate School of Medicine, 2-1-1 Hongo, Bunkyo-ku, Tokyo, 113–8421 Japan; 2 Division of Cellular Therapy/Division of Stem Cell Signaling, The Institute of Medical Science, The University of Tokyo, 4-6-1 Shirokanedai, Minato-ku, Tokyo 108-8639 Japan; 3 Analytical Science Laboratories, Kao Corporation, Haga Tochigi, 321-3497 Japan; 4 Department of Chemotherapy and Mycoses, National Institute of Infectious Diseases, 1-23-1 Toyama, Shinjuku-ku Tokyo, 162-8640 Japan; 5 Department of Hematology, Hokkaido University Graduate School of Medicine, Sapporo Hokkaido, 060-0808 Japan; 6 Department of Pediatrics and Adolescent Medicine, Juntendo University Graduate School of Medicine, Tokyo, 113-8421 Japan; 7 Departments of Gastroenterology Immunology, Juntendo University Graduate School of Medicine, Tokyo, Japan; 8 Department of Immune Regulation, Graduate School of Medical and Dental Sciences, Tokyo Medical and Dental University, Tokyo, 113-8510 Japan; 9 Department of Infection Biology, University Hospital Erlangen and Friedrich-Alexander University Erlangen-Nuremberg, Erlangen, Germany; 10 Medical Faculty “Carl-Gustav Carus”, Institute for Immunology, Technische Universität Dresden, Fetscherstraße 74, Dresden, 01307 Germany; 11 Laboratory of Systems Biology, Center for Experimental Medicine and Systems Biology, The Institute of Medical Science, The University of Tokyo, Tokyo, 108-8639 Japan

## Abstract

Sepsis is a serious clinical problem. Negative regulation of innate immunity is associated with sepsis progression, but the underlying mechanisms remains unclear. Here we show that the receptor CD300f promotes disease progression in sepsis. *CD300f* ^−/−^ mice were protected from death after cecal ligation and puncture (CLP), a murine model of septic peritonitis. CD300f was highly expressed in mast cells and recruited neutrophils in the peritoneal cavity. Analysis of mice (e.g., mast cell-deficient mice) receiving transplants of wild-type or *CD300f* ^−/−^ mast cells or neutrophils indicated that CD300f deficiency did not influence intrinsic migratory abilities of neutrophils, but enhanced neutrophil chemoattractant production (from mast cells and neutrophils) in the peritoneal cavity of CLP-operated mice, leading to robust accumulation of neutrophils which efficiently eliminated *Escherichia coli*. Ceramide-CD300f interaction suppressed the release of neutrophil chemoattractants from *Escherichia coli*-stimulated mast cells and neutrophils. Administration of the reagents that disrupted the ceramide-CD300f interaction prevented CLP-induced sepsis by stimulating neutrophil recruitment, whereas that of ceramide-containing vesicles aggravated sepsis. Extracellular concentrations of ceramides increased in the peritoneal cavity after CLP, suggesting a possible role of extracellular ceramides, CD300f ligands, in the negative-feedback suppression of innate immune responses. Thus, CD300f is an attractive target for the treatment of sepsis.

## Introduction

Septic peritonitis is a life-threatening emergency. Most clinical trials of anti-inflammatory agents in septic patients have been unsuccessful. Sepsis is characterized by systemic dysregulated inflammatory responses to infection. Despite extensive studies aimed at controlling or preventing it, sepsis remains a substantial clinical problem with poor prognosis and limited therapeutic options^1, 2^. The innate immune system is the first line of defense against bacterial infection. Tissue-resident myeloid cells―including mast cells and macrophages―detect invading bacteria or their products via pattern recognition receptors. To recruit neutrophils to infection sites, these myeloid cells release a variety of chemical mediators: neutrophil chemoattractants (e.g., leukotriene B4 [LTB4], macrophage inflammatory protein 2 [MIP2], and keratinocyte-derived chemokine [KC]) and vascular inflammation- and permeability-inducing factors (e.g., leukotrienes [LTs] and histamine). In turn, the recruited neutrophils themselves release neutrophil chemoattractants to further recruit neutrophils, which engulf and kill bacteria, thereby preventing the bacteria from spreading^3–6^. Nonetheless, once bacterial infections overcome such coordinated innate responses, sepsis progresses with systemic hyper-inflammation followed by immunosuppression. Most clinical trials of anti-inflammatory agents in septic patients have been unsuccessful^2^. Excessive activation of innate immunity is counterbalanced by negative signaling cascades^7, 8^. Therefore, understanding the inhibitory mechanisms in innate host responses is necessary to develop effective treatments of sepsis. Cecal ligation and puncture (CLP) is the standard model of septic peritonitis triggered by self-infection with intestinal bacteria such as *Escherichia coli* (*E. coli*). A variety of immune cells and receptors regulate CLP-induced sepsis during its different phases^9–11^. Here, we delineate the critical role of mast cell- and neutrophil-expressed CD300f (also called leukocyte mono-immunoglobulin-like receptor 3 [LMIR3] or CMRF-35-like molecule-1 [CLM-1])^12–15^ in innate host responses.

CD300f belongs to the paired activating and inhibitory receptor family CD300 (also called LMIR, CLM, or myeloid-associated Ig-like receptor [MAIR])^12–18^. The inhibitory receptor CD300f harbors two immunoreceptor tyrosine-based inhibitory motifs (ITIMs) and a single immunoreceptor tyrosine-based switch motif (ITSM) in the cytoplasmic region, and CD300f is expressed in myeloid cells, including mast cells and neutrophils^12–15^. Recent studies point to specific lipids or lipid-binding proteins as ligands of CD300^13, 19–26^. We recently identified extracellular ceramide as a ligand of CD300f^13^. Ceramides are composed of a long-chain or sphingoid base linked to a fattty acid via an amide bond. Ceramides play an important role not only as structural elements but also as regulators of a variety of cellular processes including differentiation, inflammation, proliferation, and apoptosis^27^. Ceramide-CD300f interaction inhibits IgE-dependent allergic responses^13^, ATP-mediated experimental colitis^28^, or lipopolysaccharide (LPS)-induced skin inflammation^29^. However, the roles of ceramide-CD300f interaction in sepsis, including septic peritonitis, have remained elusive.

In the present study, we demonstrate that in a model of septic peritonitis, disrupting the ceramide-CD300f interaction remarkably stimulates neutrophil recruitment to sites of infection and protects mice from septic death.

## Results

### *CD300f*^−/−^ mice were highly resistant to CLP-induced sepsis

**Figure 1:**
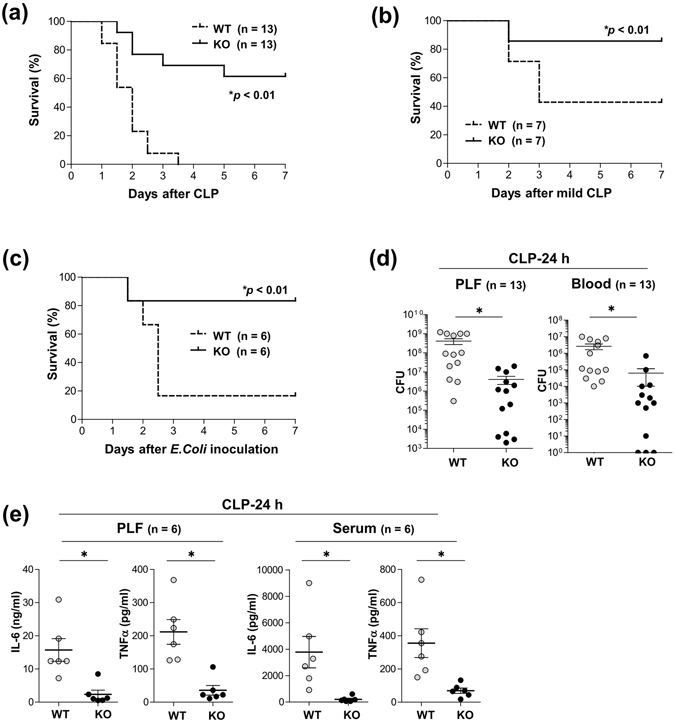
*CD300f* ^−/−^ mice were highly resistant to CLP-induced sepsis. (**a**,**b**) WT or *CD300f* ^−/−^ mice were subjected to (**a**) CLP (n = 13 per genotype) or (**b**) mild CLP (n = 7 per genotype), and monitored regarding survival. (**c**) WT or *CD300f* ^−/−^ mice (n = 6 per genotype) were intraperitoneally inoculated with a suspension of *E. coli* (4.0 × 10^8^ colony-forming units [CFU] per mouse) and monitored regarding survival. (**a**–**d**) *p < 0.01 (log-rank test). (**d**) Bacterial counts (CFUs) in PLF or PB of WT or *CD300f* ^−/−^ mice 24 h after CLP (n = 13 per genotype). (**e**) The concentrations of IL-6 or TNF-α in PLF or serum of WT or *CD300f* ^−/−^ mice 24 h after CLP (n = 6 per genotype). (**d**,**e**) The data are expressed as mean ± SD; *p < 0.01 (Student’s *t*-test). (**a**,**b**,**c** and **e**) The data are representative of two independent experiments. WT and KO indicate WT mice and *CD300f* ^−/−^ mice, respectively.

To clarify the role of CD300f in innate host responses, we used CLP, a model of septic peritonitis, in WT and *CD300f* ^−/−^ mice. *CD300f* ^−/−^ mice showed prolonged survival after CLP or mild CLP as compared with WT mice (Fig. 1a,b). Likewise, *CD300f* ^−/−^ mice were less prone to septic death after intraperitoneal inoculation with a minimal lethal dose of *E. coli* (Fig. 1c). Notably, lower counts of *E. coli* as well as remarkably lower levels of the proinflammatory cytokines tumor necrosis factor (TNF)-α and interleukin (IL)-6 were found in both peritoneal lavage fluid (PLF) and peripheral blood (PB) collected 24 h after CLP in *CD300f* ^−/−^ mice compared with WT mice (Fig. 1d,e). Thus, the vascular spread of *E. coli* and the concomitant hyper-inflammatory responses were prevented in *CD300f* ^−/−^ mice as early as 24 h after CLP, indicating that *CD300f* ^−/−^ mice were highly resistant to CLP-induced sepsis.

### Markedly enhanced accumulation of neutrophils was observed in the peritoneal cavity of *CD300f*^−/−^ mice after CLP

**Figure 2:**
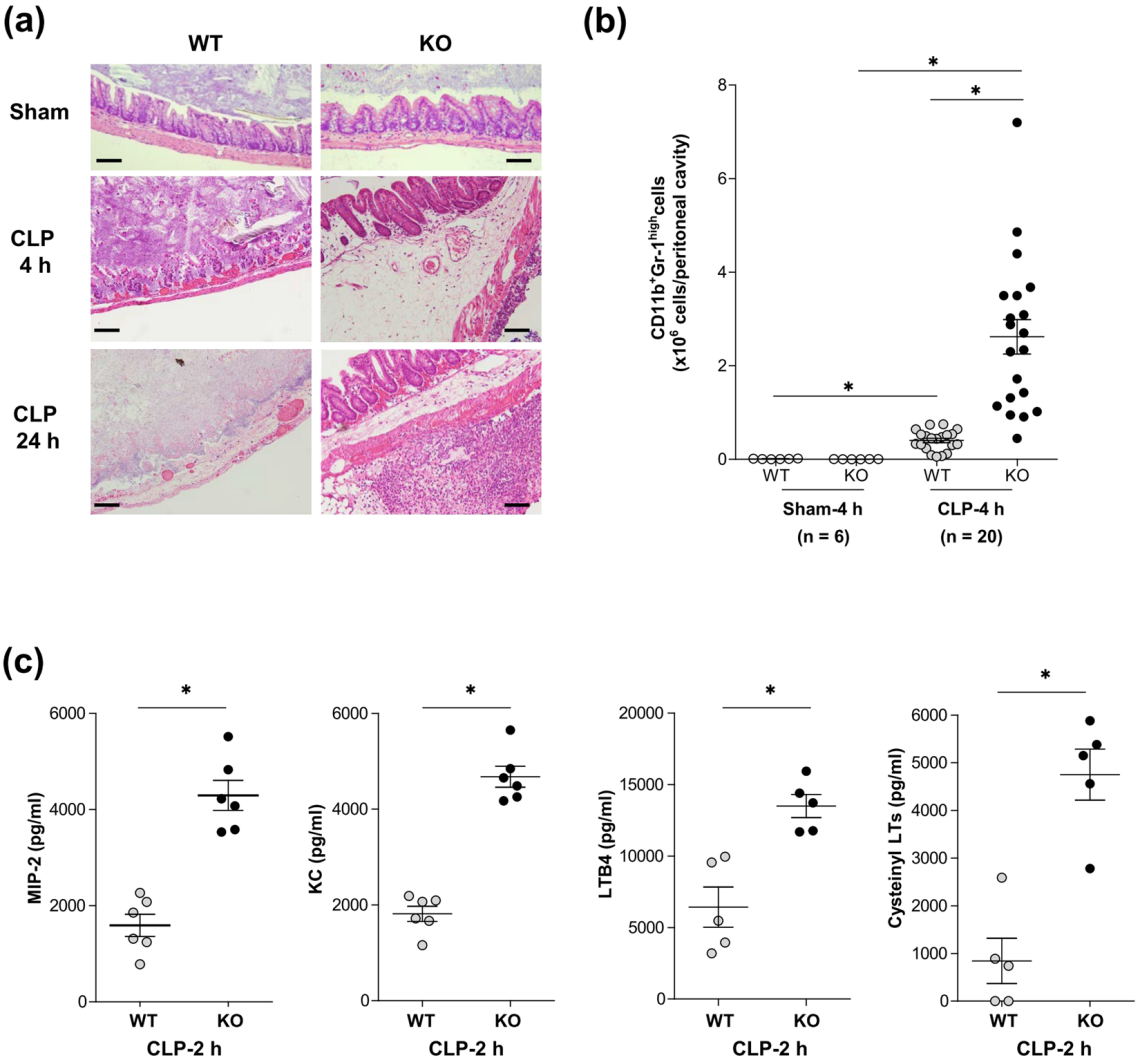
Neutrophil recruitment to infection sites was enhanced in CLP-operated *CD300f* ^−/−^ mice. (**a**) Cecum sections from WT or *CD300f* ^−/−^ mice at 4 h (top) after a sham operation and 4 h (middle) or 24 h (bottom) after CLP were stained with hematoxylin and eosin. Scale bars are 100 μm. The data are representative of five independent experiments. (**b**) Numbers of neutrophils recruited into the peritoneal cavity of the indicated mice 4 h after a sham operation (total n = 6) or CLP (total n = 20). Data were pooled from two independent experiments. (**c**) The concentrations of MIP2, KC, LTB4, or cysteinyl LTs in PLF in the indicated mice 2 h after CLP (n = 5–6 per genotype). The data are representative of three independent experiments and are expressed as mean ± SD; *p < 0.01 (Student’s *t*-test).

We then performed histological analysis of cecum sections, which showed no significant differences between WT and *CD300f* ^−/−^ mice after a sham operation (Fig. 2a). WT mice, but not *CD300f* ^−/−^ mice, showed severe destruction or a complete loss of mucosal architecture in cecum sections collected 4 or 24 h after CLP, respectively (Fig. 2a). Cecum sections of *CD300f* ^−/−^ mice revealed prominent submucosal edema and serosal neutrophil infiltration 4 h after CLP, and showed attenuated edema and enhanced neutrophil accumulation after 24 h (Fig. 2a). In line with the histological findings, we observed a marked increase in the number of CD11b^+^Gr-1^high^ neutrophils in PLF of *CD300f* ^−/−^ mice 4 h after CLP (Fig. 2b). We then measured the levels of MIP2, KC, LTB4, or cysteinyl LTs in PLF of the CLP-operated mice. The results showed higher concentrations of these chemical mediators in PLF of *CD300f* ^−/−^ mice as compared with PLF of WT mice as early as 2 h after CLP (Fig. 2c). These results led us to hypothesize that the high levels of neutrophil chemoattractants and vascular inflammation- and permeability-inducing factors recruit substantial numbers of neutrophils to the peritoneal cavity of *CD300f* ^−/−^ mice.

### Ceramide-CD300f interaction suppressed the release of neutrophil chemoattractants from mast cells and neutrophils in response to the presence of *E. coli*

**Figure 3:**
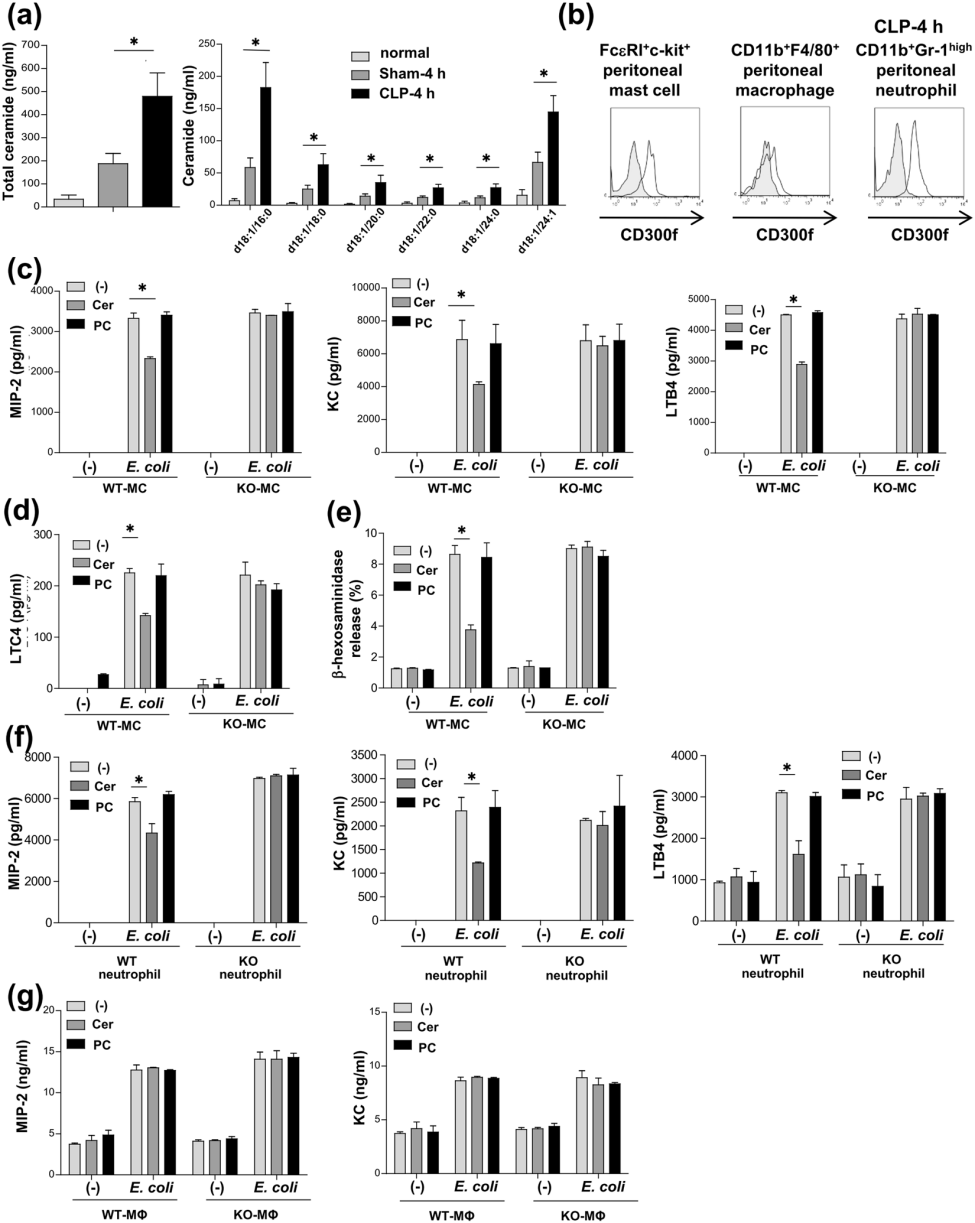
Ceramide-CD300f interaction suppressed the release of factors (inducing neutrophil migration) from mast cells and neutrophils in response to the presence of *E. coli*. (**a**) The concentrations of all and indicated ceramide species in PLF before or 4 h after a sham operation or CLP in WT mice (n = 5 per group). (**b**) Surface expression levels of CD300f in peritoneal mast cells (MC) or macrophages (Mϕ) before CLP or in neutrophils 4 h after CLP in WT mice. C-G, WT or *CD300f* ^−/−^ BMMCs (**c**–**e**) neutrophils (**f**), or peritoneal macrophages (**g**) were stimulated with 5 × 10^7^ CFU/ml heat-killed *E. coli* on plates coated with ceramide, PC, or vehicle. Production of (**c**,**f**) MIP2, KC, and LTB4, (**d**) LTC4, (**e**) a release of β-hexosaminidase, or (**f**) MIP2 and KC. (**b**–**g**) The data are representative of four independent experiments. The data are expressed as mean ± SD; *p < 0.01 (Student’s *t*-test).

To clarify the role of the interaction between CD300f and its ligand ceramide in CLP, we examined the levels of extracellular ceramide species in PLF or plasma from WT mice^30^. The results revealed that several kinds of ceramide (N-palmitoyl-D-erythro-sphingosine [d18:1/16:0], N-stearoyl-D-erythro-sphingosine [d18:1/18:0], N-arachidoyl-D-erythro-sphingosine [d18:1/20:0], N-behenoyl-D-erythro-sphingosine [d18:1/22:0], N-lignoceroyl-D-erythro-sphingosine [d18:1/24:0], and N-nervonoyl-D-erythro-sphingosine [d18:1/24:1]) were present in PLF; the levels of ceramide species in PLF, but not in plasma, increased after CLP, implying a possible role of the ceramide-CD300f binding in this phenomena (Fig. 3a and Supplementary Fig. S1). Next, to identify the cell populations responsible for the enhanced neutrophil accumulation in CLP-operated *CD300f* ^−/−^ mice, we examined CD300f expression in peritoneal myeloid cells of WT mice. Notably, CD300f was highly expressed in resident mast cells and in the recruited neutrophils, whereas it was barely expressed in peritoneal macrophages (Fig. 3b). We then tested whether the binding of ceramide to CD300f inhibits the release of neutrophil chemoattractants from these myeloid cells in response to the presence of *E. coli*. To this end, bone marrow-derived mast cells (BMMCs) were stimulated with *E. coli* on plates coated with ceramide or phosphatidylcholine (PC) as a control. The CD300f deficiency failed to alter the release of the indicated chemical mediators from the stimulated BMMCs in the absence of ceramide (Fig. 3c–e). On the other hand, the ceramide-CD300f interaction specifically inhibited the release of MIP2, KC, LTB4, LTC4, or β-hexosaminidase (a marker of degranulation) from WT BMMCs, but not from *CD300f* ^−/−^ BMMCs, in response to the presence of *E. coli* (Fig. 3c–e). Similar experiments with BMMC transfectants confirmed that the disruption of ITIMs and ITSM of CD300f attenuated the inhibition of *E. coli*-stimulated mast cell activation by the ceramide-CD300f binding (Supplementary Fig. S2a–c)^13^. Thus, the ceramide-CD300f interaction suppressed *E. coli*-stimulated mast cell activation via ITIMs and ITSM of CD300f. Similarly, the ceramide-CD300f interaction also suppressed the release of neutrophil chemoattractants from neutrophils stimulated by *E. coli* (Fig. 3f and Supplementary Fig. S3). In contrast, the release of these neutrophil chemoattractants was not inhibited by the ceramide-CD300f interaction in peritoneal macrophages, presumably due to the low levels of CD300f expression (Fig. 3g). On the other hand, neither LMIR3 expression nor the ceramide-CD300f interaction significantly influenced the bactericidal activity of neutrophils or BMMCs against *E. coli* (Supplementary Fig. S4a,b); this finding is suggestive of a limited role of the ceramide-CD300f binding in innate immune responses.

### *CD300f*^−/−^ neutrophils recruited to the peritoneal cavity contributed to enhanced neutrophil accumulation in the CLP model

**Figure 4:**
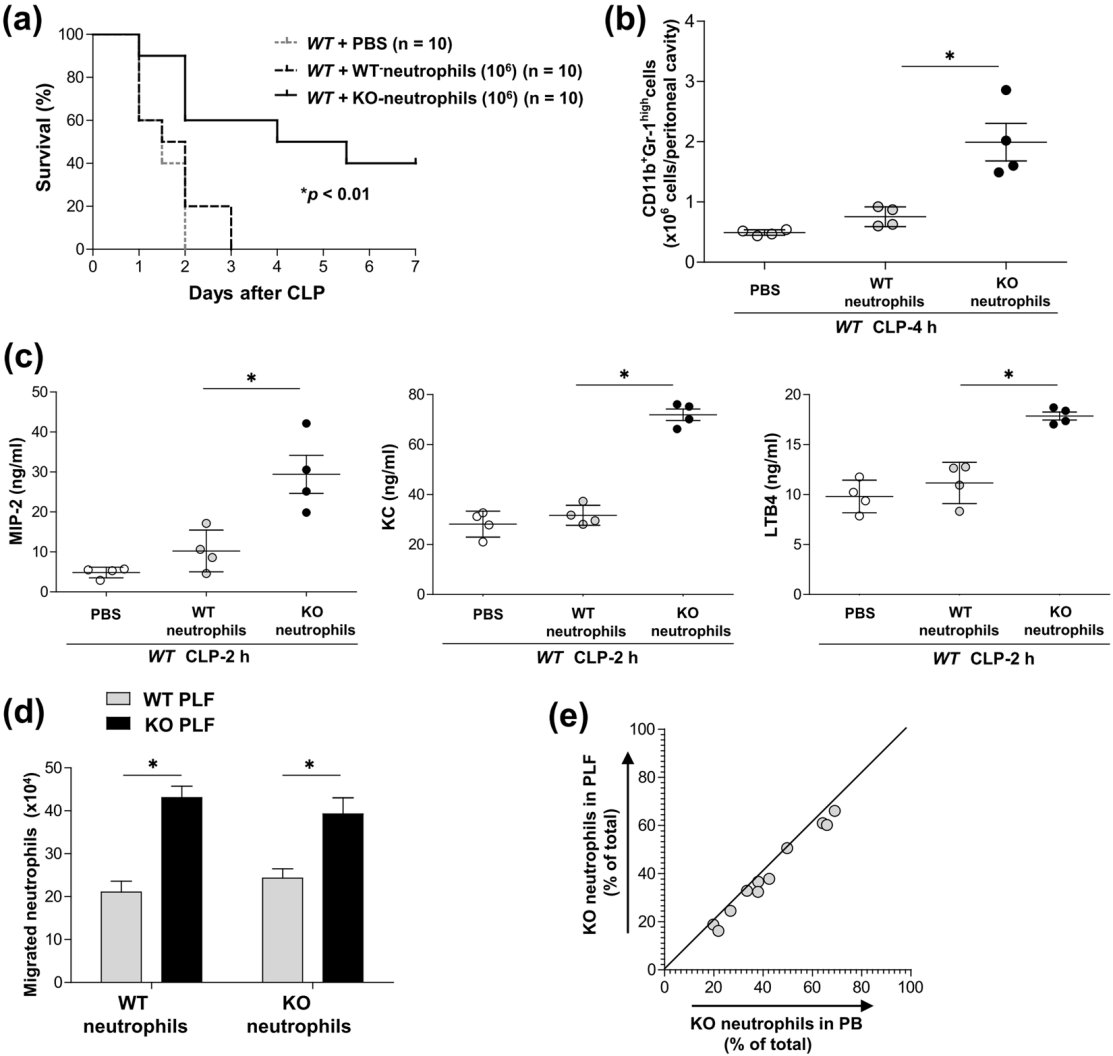
*CD300f* ^−/−^ neutrophils recruited to the peritoneal cavity contributed to enhanced neutrophil migration in a CLP model. (**a–c**) CLP-operated WT (Ly5.1^+^) mice were intraperitoneally injected with 10^6^ cells of WT (Ly5.2^+^) or *CD300f* ^−/−^ (Ly5.2^+^) neutrophils or PBS as a control (n = 6 per genotype). (**a**) The mice were monitored regarding survival; *p < 0.01 compared to the mice inoculated with WT neutrophils (log-rank test). (**b**,**c**) Numbers of Ly5.1^+^ neutrophils (**b**) or levels of MIP2, KC, or LTB4 (**c**) in PLF (each, n = 4). (**d**) Numbers of WT or *CD300f* ^−/−^ neutrophils that migrated into the lower wells containing PLF derived from either WT or *CD300f* ^−/−^ mice 4 h after CLP. (**e**) The proportions (%) of *CD300f* ^−/−^ neutrophils among CSFE-positive neutrophils present in PB or PLF from the chimeric mice (each, n = 4) 4 h after CLP. Values on the X and Y axes represent the percentage in PB and PLF, respectively. (**a–e**) The data are representative of two independent experiments. The data are expressed as mean ± SD; *p < 0.01 (Student’s *t*-test).

To clarify the *in vivo* role of CD300f-deficient neutrophils in promoting neutrophil accumulation in our CLP model, we intraperitoneally injected equal numbers of WT or *CD300f* ^−/−^ neutrophils (Ly5.2^+^) into CLP-operated WT mice (Ly5.1^+^). Transfusion of 10^7^ neutrophils, irrespective of the expression of CD300f, equally improved survival of the CLP-operated mice (Supplementary Fig. S5), suggesting that an adequate number of recruited neutrophils can prevent CLP-induced sepsis. On the other hand, transfusion of 10^6^ *CD300f* ^−/−^ neutrophils, but not of WT neutrophils, increased survival and the number of Ly5.1^+^ neutrophils recruited to (and the levels of MIP2, KC, and LTB4 in) the peritoneal cavity of the CLP-operated WT mice (Fig. 4a–c). We then determined whether the CD300f deficiency enhances the intrinsic migratory ability of neutrophils themselves. Transwell migration assays revealed that PLF of CLP-operated *CD300f* ^−/−^ mice attracted more neutrophils than did PLF of CLP-operated WT mice, and that equivalent numbers of WT or *CD300f* ^−/−^ neutrophils migrated into the same PLF (Fig. 4d). After that, to next compare the migratory ability between WT and *CD300f* ^−/−^ neutrophils *in vivo*, CFSE-labeled *CD300f* ^−/−^ neutrophils mixed in the ratio of 1:4, 1:1, or 4:1 with WT neutrophils were intravenously injected into WT mice 1 h before CLP. The proportions of *CD300f* ^−/−^ cells among CFSE-positive peritoneal neutrophils were also similar to their proportions among the CFSE-positive PB neutrophils (Fig. 4e). These data indicated that neutrophil chemoattractants released by *CD300f* ^−/−^ neutrophils rather than the intrinsic migratory ability of *CD300f* ^−/−^ neutrophils contributed to the enhanced accumulation of neutrophils in CLP-operated *CD300f* ^−/−^ mice.

### *CD300f*^−/−^ mast cells played an important role in the enhanced neutrophil accumulation in the CLP model

**Figure 5:**
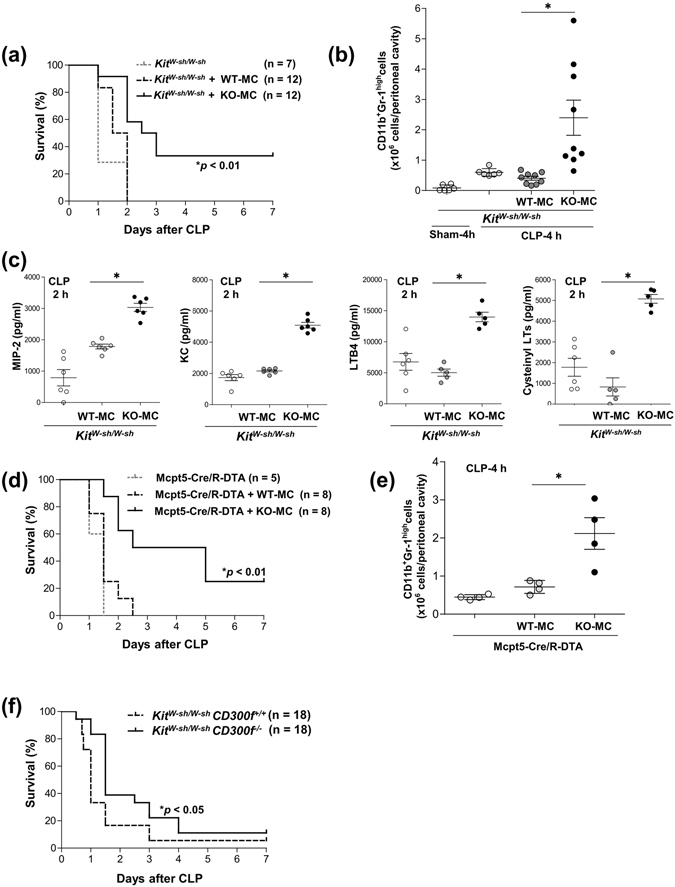
*CD300f* ^−/−^ mast cells played an important role in enhanced accumulation of neutrophils in the CLP model. (**a**) *Kit* ^*W-sh/W-sh*^ mice that had received an intraperitoneal transplant of WT or *CD300f* ^−/−^ BMMCs (n = 12 per group) or were injected with PBS (control, n = 7) were subjected to CLP and monitored regarding survival; *p < 0.01 compared to the mice transplanted with WT BMMCs (log-rank test). (**b,c**) Numbers of neutrophils recruited into the peritoneal cavity (**b**) and concentrations of MIP2, KC, LTB4, or cysteinyl LTs (**c**) in the indicated mice (n = 5 to 9 per group) 4 h after CLP. (**d**,**e**) *Mcpt5-Cre/R-DTA* mice that had received an intraperitoneal transplant of WT or *CD300f* ^−/−^ BMMCs (n = 8 per group) or were injected with PBS (control, n = 5) were subjected to CLP and monitored regarding survival. *p < 0.01 compared to the mice with a transplant of WT BMMCs (long-rank test). (**e**) Numbers of neutrophils recruited into the peritoneal cavity of the indicated mice (n = 4 per group) 4 h after CLP. (**a–e**) The data are representative of two independent experiments. (**b,c,e**) The data are expressed as mean ± SD; *p < 0.01 (Student’s *t*-test). (**f**) *Kit* ^*W-sh/W-sh*^ or *Kit* ^*W-sh/W-sh*^ *CD300f* ^−/−^ mice (total n = 18 per group) were subjected to CLP model and monitored regarding survival; *p < 0.05 (log-rank test). Data were pooled from two independent experiments.

To clarify the functions of *CD300f* ^−/−^ mast cells in the defense against sepsis, we used CLP in mast cell-deficient (*Kit* ^*W-sh/W-sh*^) mice reconstituted intraperitoneally with WT or *CD300f* ^−/−^ BMMCs, with the two types of mice having equivalent numbers of peritoneal mast cells (Supplementary Fig. S6a,b). The reconstitution with *CD300f* ^−/−^ BMMCs, but not with WT BMMCs, improved the survival of CLP-operated *Kit* ^*W-sh/W-sh*^ mice (Fig. 5a), implicating *CD300f* ^−/−^ mast cells in the prolonged survival of the CLP-operated *CD300f* ^−/−^ mice. In addition, *CD300f* ^−/−^ BMMC-reconstituted *Kit* ^*W-sh/W-sh*^ mice were found to have greater numbers of neutrophils as well as higher levels of MIP2, KC, LTB4, and cysteinyl LTs in PLF than did WT BMMC-reconstituted *Kit* ^*W-sh/W-sh*^ mice (Fig. 5b,c). Similarly, a transplant of *CD300f* ^−/−^ BMMCs, but not WT BMMCs, enhanced neutrophil accumulation and improved survival after CLP in a cross of *Mcpt5-Cre* mice with the *R-DTA* mice (*Mcpt5-Cre/R-DTA* mice); these mice were genetically engineered to be mast cell-deficient (Fig. 5d,e and Supplementary Fig. S7)^31^. Moreover, we compared disease progression between *Kit* ^*W-sh/W-sh*^ mice and *Kit* ^*W-sh/W-sh*^ *CD300f* ^−/−^ mice. The latter mice showed slightly increased survival after CLP as compared with the former (Fig. 5f), indicating that *CD300f* ^−/−^ myeloid cells other than mast cells, possibly neutrophils, contributed to the resistance of *CD300f* ^−/−^ mice to CLP lethality. Moreover, reconstitution with *CD300f* ^−/−^ BMMCs, but not with their WT counterparts, increased the number of neutrophils in the peritoneal cavity and improved survival in CLP-operated *CD300f* ^−/−^ *Kit* ^*W-sh/W-sh*^ mice (Supplementary Fig. S8a–c). On the other hand, a transplant of BM-derived macrophages, irrespective of CD300f expression, equally improved survival after CLP in WT mice with macrophage/monocyte depletion by clodronate liposomes (Supplementary Fig. S9), confirming that monocytes/macrophages play a role in the resistance to CLP-induced sepsis; however, CD300f deficiency in monocytes/macrophages does not significantly influence that.

### Treatment with an anti-ceramide Ab or CD300f-Fc ameliorated septic mortality after CLP

**Figure 6:**
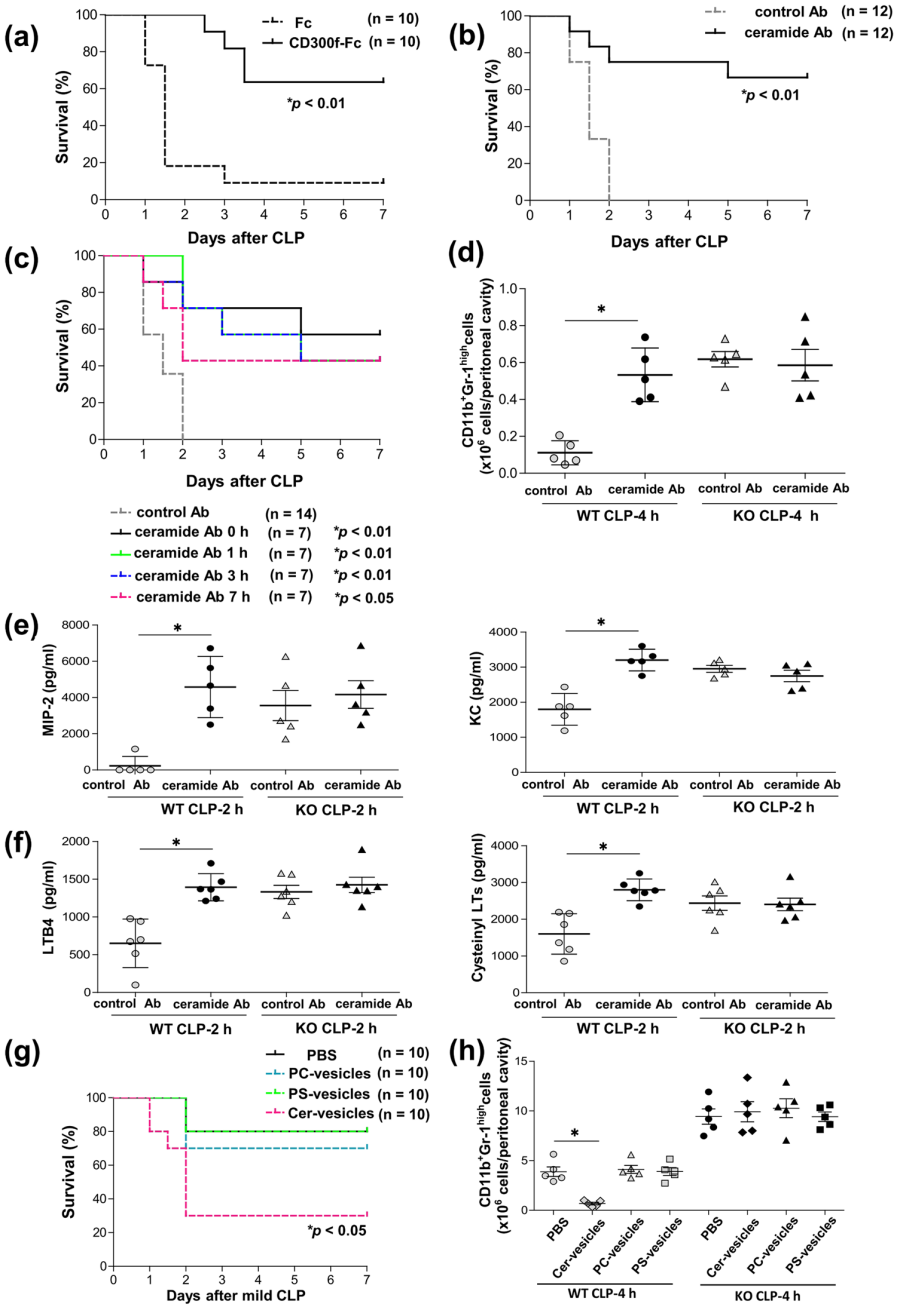
Treatment with an anti-ceramide Ab or CD300f-Fc ameliorated septic mortality after CLP. (**a**,**b**) Ab- or Fc protein-treated WT mice were subjected to CLP and monitored regarding survival. (**a**) The mice were intraperitoneally injected with 300 μg of either CD300f-Fc or control Fc immediately after CLP (n = 10); *p < 0.01 (log-rank test). (**b**) The mice were intraperitoneally injected with 4 μg of either the anti-ceramide Ab or isotype control Ab immediately after CLP (n = 12); *p < 0.01 (log-rank test). (**c**) Mice were intraperitoneally injected with 4 μg of either anti-ceramide Ab at 0, 1, 3, or 7 h after CLP (n = 7) or isotype control Ab at 0 h after CLP (n = 14); *p < 0.01 or **p < 0.05 compared to control mice (log-rank test). (**d–f**) WT or *CD300f* ^−/−^ mice were intraperitoneally injected with 4 μg of either the anti-ceramide Ab or isotype control Ab immediately after CLP. (**d**) Numbers of neutrophils recruited into the peritoneal cavity 4 h after CLP (n = 5). (**e**,**f**) Concentrations of (**e**) MIP-2, KC, or LTB4 or (**f**) cysteinyl LTs in PLF from mice 4 h after CLP (n = 5 to 6). (**g**,**h**) WT or *CD300f* ^−/−^ mice were intraperitoneally injected with 100 μg of vesicle containing ceramide, PC, PS, or PBS as a control immediately after CLP. (**g**) Mice (total n = 10 per group) were monitored regarding survival; *p < 0.05 compared to control mice (log-rank test). Data were pooled from two independent experiments. (**h**) Numbers of neutrophils recruited into the peritoneal cavity 4 h after CLP (n = 5). (**d,e,f,h**) The data are representative of two independent experiments. The data are expressed as mean ± SD; *p < 0.01 (Student’s *t*-test).

We then intraperitoneally injected CD300f-Fc or a control Fc into mice immediately after CLP and monitored survival. The CD300f-Fc-treated mice survived remarkably longer after CLP, as compared with control mice (Fig. 6a). Similarly, intraperitoneal injection of an anti-ceramide Ab, but not of a control Ab, into WT mice immediately after CLP effectively prevented septic deaths (Fig. 6b). WT mice given an injection of the anti-ceramide Ab at 1 or 3 h after CLP showed survival rates that were similar to those of the mice treated with the anti-ceramide Ab immediately after CLP (Fig. 6c). In addition, even when WT mice received an intraperitoneal injection of the anti-ceramide Ab at 7 h after CLP, this treatment was still effective against septic deaths (Fig. 6c). Accordingly, treatment with the anti-ceramide Ab, but not with a control Ab, increased the numbers of neutrophils recruited to the peritoneal cavity of WT mice 2 h after CLP; these numbers were comparable to those found in the case of CLP-operated *CD300f* ^−/−^ mice (Fig. 6d). In addition, treatment with the anti-ceramide Ab, but not with a control Ab, elevated the levels of MIP-2, KC, LTB4, and cysteinyl LTs in PLF of WT mice 2 h after CLP (Fig. 6e,f). In contrast, the same treatment failed to influence neutrophil recruitment or chemical-mediator production in CLP-operated *CD300f* ^−/−^ mice (Fig. 6d–f). In contrast, treatment with vesicle containing ceramide, but not PC or phosphatidylserine (PS), decreased the survival of WT mice after mild CLP (Fig. 6g). In line with this finding, only treatment with ceramide-containing vesicle decreased the number of neutrophils recruited to the peritoneal cavity of WT mice, but not of *CD300f* ^−/−^ mice (Fig. 6h). Thus, increasing the concentration of extracellular ceramides aggravated experimental sepsis by reducing neutrophil recruitment to the local sites of infection, whereas disruption of the ceramide-CD300f interaction prevented sepsis by stimulating neutrophil recruitment.

## Discussion

A striking feature of *CD300f* ^−/−^ mice subjected to CLP is the dramatically enhanced accumulation of neutrophils after elevated production of neutrophil chemoattractants (e.g., MIP2, KC, and LTB4) and vascular permeability- and inflammation-inducing factors (e.g., cysteinyl LTs) in the peritoneal cavity, where feces**-**derived bacteria (e.g., *E. coli*) evoked inflammation. Given that CD300f deficiency enhances LPS-induced skin inflammation characterized by edema formation and neutrophil accumulation^29^, it is possible that *E. coli*-derived LPS plays a certain role in the underlying pathogenesis. Notably, peritoneal exudates from CLP-operated *CD300f* ^−/−^ mice attracted more neutrophils, irrespective of CD300f expression, than did those from WT counterparts in an *in vitro* migration assay. In addition, WT and CD300f-deficient neutrophils had equivalent abilities to migrate from the circulating blood to the peritoneal cavity in our CLP model. Therefore, it was reasonable to assume that the enhanced neutrophil accumulation in *CD300f* ^−/−^ mice was primarily due to elevated local production of factors inducing neutrophil migration at an early time-point after CLP; peritoneal *CD300f* ^−/−^ myeloid cells should contribute to this phenomenon. On the other hand, flow cytometric analysis revealed high levels of CD300f expression in peritoneal mast cells and in recruited neutrophils, but not in peritoneal macrophages. Consistent with this, the binding of plate-coated ceramide to CD300f inhibited the release of factors (inducing neutrophil migration) from mast cells and neutrophils, but not from peritoneal macrophages, in response to the presence of *E. coli*. Accordingly, we hypothesized that the ceramide-CD300f binding in resident mast cells and recruited neutrophils would suppress the local production of factors inducing neutrophil migration in the CLP model. Indeed, we confirmed that ceramide species were present in PLF. Moreover, CLP substantially increased the amounts of peritoneal ceramide species; this finding is suggestive of a possible role of extracellular ceramides in the negative-feedback suppression of innate immune responses^32^. Expression patterns of ceramide species were different between plasma and PLF, implying that extracellular ceramides may be derived from damaged, necrotic, or apoptotic cells, but not from extravasated blood, in the peritoneal cavity after CLP. Moreover, treatment with CD300f-Fc or the anti-ceramide Ab, which disrupt the ceramide-CD300f interaction *in vivo* ^13^, increased the number of neutrophils recruited to and the levels of neutrophil chemoattractants in the peritoneal cavity of WT mice. In contrast, treatment with ceramide-containing vesicle, which locally upregulated ligands for CD300f^13^, decreased neutrophil accumulation and the concentrations of the above mentioned mediators in WT mice. All these observations support our hypothesis that the ceramide-CD300f interaction suppresses innate immune responses in the CLP model.

Inoculation of an adequate number (10^7^) of either WT or *CD300f* ^−/−^ neutrophils equally improved survival of WT mice after CLP, suggesting that robust accumulation of neutrophils prevented sepsis after CLP in *CD300f* ^−/−^ mice. On the other hand, inoculation of low numbers (10^6^) of *CD300f* ^−/−^ neutrophils, but not WT neutrophils, increased the number of recipient neutrophils recruited to the peritoneal cavity, concentrations of neutrophil chemoattractants in PLF, and survival after CLP in WT mice. In addition, *CD300f* ^−/−^ *Kit* ^*W-sh/W-sh*^ mice were less prone to CLP-induced death than *Kit* ^*W-sh/W-sh*^ mice were. These results implicated *CD300f* ^−/−^ neutrophils in the resistance of *CD300f* ^−/−^ mice to septic death. We can theorize that once recruited to inflamed tissues containing extracellular ceramides, *CD300f* ^−/−^ neutrophils release more neutrophil chemoattractants than WT neutrophils do, thus accelerating neutrophil accumulation in the CLP model.

The crucial role of CD300f-deficient mast cells in CLP-operated *CD300f* ^−/−^ mice was illustrated by several lines of evidence: the concentrations of neutrophil chemoattractants and cysteinyl LTs in PLF, the number of neutrophils recruited to the peritoneal cavity, and survival after CLP were increased by implantation of *CD300f* ^−/−^ mast cells, but not WT mast cells, in two types of mast cell-deficient mice (*Kit* ^*W-sh/W-sh*^ or *Mcpt5-Cre/R-DTA*). In addition, similar results were obtained in *CD300f* ^−/−^ *Kit* ^*W-sh/W-sh*^ mice, confirming the non-redundant and indispensable role of *CD300f* ^−/−^ mast cells in this model. Recent studies suggested that whether mast cells have a beneficial or detrimental effect on survival during bacterial infection depends on the circumstances, including the type and severity of infection^33, 34^. In fact, under our experimental conditions, WT mast cells appeared to not promote survival after CLP. It was evident, however, that *CD300f* ^−/−^ mast cells drive neutrophil recruitment and enhance survival after CLP; these data are suggestive of an outstanding role of *CD300f* ^−/−^ mast cells in prevention of sepsis. Prominent mucosal edema in the cecum of CLP-operated *CD300f* ^−/−^ mice can be attributed to upregulation of factors increasing vascular permeability, including cysteinyl LTs, which are released mainly by mast cells. On the other hand, the increased concentrations of mast cell-derived chemical mediators known to improve survival after CLP^35, 36^ may also contribute to the resistance of *CD300f* ^−/−^ mice to septic peritonitis.

Collectively, these results support our hypothesis that resident mast cells collaborate with the recruited neutrophils to accelerate neutrophil accumulation at sites of infection in *CD300f* ^−/−^ mice although a contributory role of other *CD300f* ^−/−^ myeloid cells cannot be ruled out. On the other hand, the ceramide-CD300f binding failed to significantly influence the *in vitro* bactericidal abilities of mast cells or neutrophils in response to the presence of *E. coli*. Hence, we can conclude that the resistance of *CD300f* ^−/−^ mice to CLP lethality is likely to the result of efficient elimination of *E. coli* by vast numbers of neutrophils recruited to the peritoneal cavity where large amounts of the factors inducing neutrophil recruitment were released by resident mast cells and by recruited neutrophils.

CD300f was reported to recognize PS^21, 25^, but our results did not implicate this interaction in the CD300f-mediated inhibition of innate immune responses in our CLP model. This conclusion seems to be supported by a recent finding that apoptotic cell-mediated inhibition of cytokine and chemokine production in CLP-operated mice depends on the PS-CD300a interaction^22^. It was therefore possible to assume that the ceramide-CD300f interaction cooperated with the PS-CD300a interaction to inhibit innate immune responses in our CLP model. Thus, the ceramide-CD300f binding inhibits CLP-induced neutrophil accumulation to sites of infection as well as IgE-dependent allergic reactions, ATP-mediated experimental colitis, or LPS-induced skin inflammation; however, the ceramide-CD300f binding is either detrimental or beneficial to human health in the former and latter case, respectively^13^.

It is worth mentioning that even post-CLP treatment with the anti-ceramide Ab improved the survival of the septic mice. Although most of the anti-inflammatory therapies for sepsis have failed in clinical trials, novel strategies to relieve immunosuppression in sepsis are emerging^2^. In this sense, CD300f is a promising target because blocking of CD300f function stimulates innate host responses that can overcome both primary bacterial infections and secondary cosmic infections. Because human CD300f binds both ceramide and sphingomyelin^20^, a novel drug specifically disrupting this binding may be effective against bacterial infections. In addition, it is possible that combined targeted therapies will synergistically improve the survival rates of septic patients.

In conclusion, ceramide-CD300f interaction inhibits innate host responses in a model of septic peritonitis. Disruption of this interaction stimulates neutrophil accumulation at infection sites. Accordingly, the development of an intervention involving CD300f seems to be an appealing approach to the prophylaxis of bacterial sepsis.

## Methods

### Mice

C57BL/6 J (Ly5.1^+^), C57BL/6 J (Ly5.2^+^), *CD300f* ^−/−^, *Kit* ^*w-sh/w-sh*^, *Kit* ^*w-sh/w-sh*^ *CD300f* ^−/−^, and *Mcpt5-Cre/R-DTA* mice were used in this study^13, 31^.

### Ethics statement

All animal experiments were approved by the ethical committee of the University of Tokyo (approval no 20–8) and Juntendo University (approval no 270015). All the methods were carried out in accordance with the approved guidelines and regulations.

### Antibodies (Abs) and other reagents

The following Abs were used: rat anti-LMIR3/CD300f (3–14–11) was obtained from ACTGen; anti-Flag (M2) and mouse IgG1 (MOPC21) were purchased from Sigma-Aldrich; fluorescein isothiocyanate (FITC)-conjugated anti-CD11b (M1/70), Gr-1 (RB6-8C5), F4/80 (BM8), or the high-affinity IgE receptor (FcεRI)-α (MAR-1), phycoerythrin (PE)-conjugated anti-Gr-1 (RB6-8C5), CD45.1 (A20), CD45.2 (104), Ly-6G (1A8), CD11b (M1/70), or c-kit (2B8) or streptavidin- or allophycocyanin (APC)-conjugated anti-c-kit (2B8) and rat IgG2a (eBR2a) were from eBioscience; anti-ceramide Ab (MID 15B4) was from Enzo Life Sciences, mouse IgM (MOPC-104E) was from BioLegend, rat IgG1/2a (G28-5) was from BD Biosciences. Clodronate liposomes were from Sigma-Aldrich. All the cytokines were obtained from R&D Systems. 1,2-Dipalmitoyl-sn-glycero-3-phosphocholine (phosphatidylcholine [PC]) and 1,2-Dipalmitoyl-sn-glycero-3-phosphoserine (phosphatidylserine [PS]) were from Echelon Biosciences Inc. All other reagents were from Sigma unless stated otherwise^13^.

### Cells

BMMCs and BMMC transfectants were generated as described elsewhere^13, 37^. Neutrophils were isolated from mouse BM by means of a three-layer gradient as described previously^12, 38, 39^. Peritoneal macrophages and BM-derived macrophages were isolated as described elsewhere^17, 26^.

### Cell stimulation

Lipids were diluted to a concentration of 20 μg/ml in methanol. MaxiSorp 96-well plates (Nunc, catalog No. 430341) were coated with 50 μl of each solution, air-dried, and washed twice with the medium, as previously described^13^. BMMCs, neutrophils, or peritoneal macrophages were pre-incubated on a lipid-coated plate for 1 h before stimulation with 5 × 10^7^ CFU/ml heat-killed *E. coli* ^13^.

### Peritonitis induced by CLP or by inoculation of *E. coli*

CLP was performed as previously described^10, 11, 40–42^. Briefly, male mice were anesthetized, and the cecum was exposed through a 1.0- to 1.5-cm abdominal midline incision. The cecum was ligated at half the distance between the distal pole and the base of the cecum, followed by a single puncture with either a G-18 needle (CLP with 80–100% lethality among wild-type [WT] mice) or a G-22 needle (mild CLP with 40–60% lethality among WT mice). Sham-operated mice underwent an identical laparotomy without CLP. The operated mice received a subcutaneous injection of sterile saline (1 ml) for fluid resuscitation after the abdominal incision was closed. Survival after CLP was assessed every 12 h for 7 d. Peritoneal lavage fluid (PLF) (1 ml PBS), serum, or cecum samples were collected from the mice euthanized 2, 4, or 24 h after CLP. In some experiments, 4 μg of either the anti-ceramide Ab or isotype control Ab or 300 μg of either CD300f-Fc or control Fc was intraperitoneally injected immediately after or at the indicated time points after CLP. To induce *E. coli* peritonitis, *E. coli* was isolated from mouse feces with Drigalski agar (Nissui), was grown in Luria-Bertani broth overnight, washed with PBS four times, and re-suspended in PBS (10^9^ colony forming unit [CFU]/ml)^1, 40, 43^. Female mice were intraperitoneally injected with a suspension of *E. coli* (4.0 × 10^8^ CFU per mouse as the minimum lethal dose). For some experiments, *E. coli* was killed by heat (95 °C for 2 min)^33^.

### Analysis of *in vivo* neutrophil migration

Carboxyfluorescein diacetate succinimidyl ester (CFSE)-labeled *CD300f* ^−/−^ (Ly5.2^+^) neutrophils mixed with WT (Ly5.1^+^) neutrophils were intravenously injected into WT (Ly5.1^+^) mice 1 h before CLP. The peritoneal lavage cells or PB cells 4 h after CLP were stained with PE-conjugated anti-Ly5.1 (CD45.1) Ab or ant-Ly5.2 (CD45.2) Ab, and the proportions of *CD300f* ^−/−^ (Ly5.2^+^) cells among CFSE-positive neutrophils recruited to the peritoneal cavity or among CFSE-positive PB neutrophils were calculated by means of fluorescence-activated cell sorting (FACS).

### Preparation of lipid-containing vesicle

After 1 mg of a dry lipid (C-24 ceramide, PC, or PS) was hydrated with 1 ml of PBS, vesicle was generated by means of Avanti Mini-Etruder (Avanti Polar Lipids, Inc.) according to the manufacturer’s instructions, as previously described^13, 20^.

### Preparation of Fc fusion proteins

These proteins were purified as described^1, 7^. Endotoxin levels of Fc fusion proteins were less than 0.01 ng/μg protein as determined by the limulus amebocyte lysate test (Lonza)^13, 18^.

### Quantification of cytokines, chemokines, and LTs and the degranulation assay

Enzyme-linked immunosorbent assay (ELISA) kits for IL-6, TNF-α, KC, MIP-2, and LTB4 (R&D Systems) or cysteinyl LTs and LTC4 (Cayman Chemical Company) were used. The release of β-hexosaminidase was estimated as described elsewhere^13^.

### Flow cytometry

Flow cytometric analysis was performed on a FACSCalibur (BD Biosciences) equipped with the CellQuest software and FlowJo software (Tree Star), as previously described^13^.

### DNA constructs

pMXs-internal ribosome entry sites (IRES)-puro^r^ (pMXs-IP), pMXs-Flag-CD300f or CD300f-Y241F/Y289F/Y325F-IP, and the pME18S-hIgG1 Fc vector (a kind gift from H. Arase, Osaka University) were described previously^13, 44, 45^.

### Transfection and infection

Retroviral transfection was performed as described elsewhere^13, 44, 45^. Cells were infected with retroviruses generated by transient transfection of PLAT-E packaging cells.

### Reconstitution with BMMC

Mast cell reconstitution was performed as previously described^13^. In brief, *Kit* ^*W-sh/W-sh*^ mice or *Mcpt5-Cre/R-DTA* mice were injected intraperitoneally with either 5 × 10^6^ WT or *CD300f* ^−/−^ BMMCs 6 weeks before CLP. Reconstitution of mast cells was confirmed by toluidine blue staining or fluorescence-activated cell sorting (FACS).

### Macrophage reconstitution

Macrophage reconstitution was performed as previously described^46^. Briefly, clodronate liposomes (50 mg in 200 μl per mouse) were intraperitoneally injected into WT mice to deplete macrophages. Two days later, 10^7^ WT or *CD300f* ^−/−^ BM-derived macrophages were intraperitoneally injected into macrophage-depleted mice 6 h before CLP induction.

### Transwell migration assays

These assays were performed using Transwell filters with 3-μm pores (BD Falcon), as previously described^26^. Briefly, the upper wells contained 1.5 × 10^6^ cells in 0.2 ml of the medium and the lower wells were filled with 0.6 ml of PLF from CLP-operated mice. After incubation for 60 min, neutrophils that migrated into the lower wells were counted.

### Killing assays

The indicated cells were incubated with *E. coli* (4 × 10^5^ cells per 10^6^ CFU of *E. coli*) for 60 min. Enumeration of viable *E. coli* cells involved colony-counting methods^40, 42^.

### Quantitation of ceramides

Concentrations of ceramides were measured as previously described^30^. Briefly, PLF (1 ml PBS) or plasma samples were collected from WT mice before or 4 h after a sham or CLP operation. Total lipids were extracted by the method of Bligh and Dyer. The lipid extracts were dissolved in chloroform:methanol:2-propanol (10:45:45, v/v/v) so that the final concentration of the internal standard was 50 nM. This lipid solution was subjected to reversed-phase liquid chromatography with mass spectrometry (LC/MS). An Agilent 1100 Series LC/MSD SL system equipped with a multi-ion source, ChemStation software, a 1,100-well plate autosampler (Agilent Technologies), and an L-column ODS (2.1 mm i.d. ×150 mm; Chemicals Evaluation and Research Institute) was used. Chromatographic separation of the lipids was attained at a flow rate of 0.2 mL/min using a binary gradient solvent system of mobile phases. Each ceramide species was detected by selected ion monitoring as *m/z* [M + CH_3_COO]^−^. C24-ceramide (d18:1/24:0), C18-ceramide (d18:1/18:0), C16-ceramide (d18:1/16:0) (Toronto Research Chemicals Inc.), N-Nervonoyl-D-erythro-sphingosine (d18:1/24:1) (Cayman Chemical Co.), N-Behenoyl-D-erythro-sphingosine (d18:1/22:0) (Avanti Polar Lipids), and N-Arachidoyl-D-erythro-Sphingosine (d18:1/20:0) (Avanti Polar Lipids) served as standards. N-Heptadecanoyl-D-erythro-sphingosine (d18:1/17:0) (Avanti Polar Lipids) was used as an internal control.

### Statistical analyses

The results are expressed as means ± standard deviation (SD). Unpaired Student’s *t* test was used to analyze the differences between groups. The Kaplan-Meier method and log-rank tests were used to analyze the survival data. *p < 0.01 or **p < 0.05 was assumed to mean statistical significance.

## Electronic supplementary material

Supplemntal figures and figure legends
