# Supplementary material for: Disrupting ceramide-CD300f interaction prevents septic peritonitis by stimulating neutrophil recruitment
Source: Sci Rep. 2017 Jun 27;7:4298. doi: 10.1038/s41598-017-04647-z (PMC5487349; doi:10.1038/s41598-017-04647-z)

Supplementary information

Disrupting ceramide-CD300f interaction prevents septic peritonitis by stimulating neutrophil recruitment

Kumi Izawa[1,2], Akie Maehara[1,2], Masamichi Isobe[1,2], Yuka Yasuda[3], Makoto Urai[4], Yasutaka Hoshino[4], Keigo Ueno[4], Toshihiro Matsukawa[2,5], Mariko Takahashi[2], Ayako Kaitani[1,2], Emiko Shiba[1,6], Ayako Takamori[1], Shino Uchida[1,7], Koichiro Uchida[1], Keiko Maeda[1], Nobuhiro Nakano[1], Yoshinori Yamanishi[2,8], Toshihiko Oki[2], David Voehringer[9], Axel Roers[10], Susumu Nakae[11], Junko Ishikawa[3], Yuki Kinjo[4], Toshiaki Shimizu[1,6], Hideoki Ogawa[1], Ko Okumura[1], Toshio Kitamura[2] & Jiro Kitaura[1,2]

Supplementary figure legends S1-S9

Figure S1. Concentrations of all and indicated ceramide species in plasma. Concentrations of all and indicated ceramide species in plasma before (n =3) or 4 h after a sham operation (n = 4) or CLP (n = 4) in WT mice. The data are expressed as mean ± SD.

Figure S2. Disruption of ITIMs and ITSM of CD300f attenuated the inhibition of *E. coli*-stimulated mast cell activation. (**a**) Expression of c-Kit, FcεRIα, or CD300f in *CD300f*$^{-/-}$ BMMCs transduced with CD300f WT, CD300f-Y241F/Y289F/Y325F mutant, or mock. (**b,c**) These BMMC transfectants were stimulated with 5 x 10^7 CFU/ml heat-killed *E. coli* on plates coated with ceramide, PC, or vehicle. Production of (**b**) the release of β-hexosaminidase or (**c**) KC. The data are representative of three independent experiments and are expressed as mean ± SD. **P* < 0.01 (Student's *t*-test).

Figure S3. Expression of CD11b, Gr-1, and CD300f in WT or *CD300f*$^{-/-}$ neutrophils. Neutrophils purified from WT or *CD300f*$^{-/-}$ BM were stained with an anti-CD11b Ab, anti-Gr-1 Ab, or anti-CD300f Ab. The data are representative of three independent experiments.

Figure S4. Neither CD300f expression nor ceramide-CD300f interaction significantly influenced bactericidal activity of neutrophils or BMMCs against *E. coli.* (**a,b**) Neutrophils (**a**) or (**b**) BMMCs were incubated with *E. coli* (4 x 10^5 cells per 10^6 CFU/ml) for 60 min on plates coated with the indicated lipids or vehicle. Numbers

of viable *E. coli* cells were estimated by colony-counting methods. The data are representative of three independent experiments and are expressed as mean ± SD.

Figure S5. Transfusion of 10^7 WT or *CD300f*$^{-/-}$ neutrophils equally improved the survival of CLP-operated mice. CLP-operated WT mice were intraperitoneally injected with 10^7 WT or *CD300f*$^{-/-}$ neutrophils or PBS as a control (n = 6 per group) and were monitored regarding survival. $*p < 0.01$ compared to CLP-operated control mice; n.s.: not significant (long-rank test).

Figure S6. *Kit*$^{W\text{-}sh/W\text{-}sh}$ mice reconstituted intraperitoneally with WT or *CD300f*$^{-/-}$ BMMCs had equivalent numbers of peritoneal mast cells. (**a**) Peritoneal lavage cells from *Kit*$^{W\text{-}sh/W\text{-}sh}$ mice that received a transplant of WT or *CD300f*$^{-/-}$ BMMCs were stained for FcεRI and c-kit to determine the percentage of mast cells among peritoneal lavage cells (upper). Surface expression levels of CD300f in FcεRI$^+$c-kit$^+$ mast cell populations of peritoneal lavage cells (lower). (**b**) Numbers of mast cells among peritoneal lavage cells from *Kit*$^{W\text{-}sh/W\text{-}sh}$ mice that received a transplant of WT or *CD300f*$^{-/-}$ BMMCs (each, n = 7). The data are representative of two independent experiments. The data are expressed as mean ± SD.

Figure S7. *Mcpt5-Cre/R-DTA* mice reconstituted intraperitoneally with WT or *CD300f*$^{-/-}$ BMMCs had equivalent numbers of peritoneal mast cells. Numbers of mast cells among peritoneal lavage cells from *Mcpt5-Cre/R-DTA* mice with a transplant of WT or *CD300f*$^{-/-}$ BMMCs (each, n = 5). The data are representative of two independent experiments and are expressed as mean and ± SD.

Figure S8. Reconstitution with *CD300f*$^{-/-}$ BMMCs increased the number of neutrophils recruited to the peritoneal cavity and improved survival in CLP-operated *Kit*$^{W-sh/W-sh}$*CD300f*$^{-/-}$ mice. (**a**) *Kit*$^{W-sh/W-sh}$*CD300f*$^{-/-}$ mice that had received an intraperitoneal transplant of WT or *CD300f*$^{-/-}$ BMMCs (n = 11 per genotype) or were injected with PBS (n = 3) were subjected to CLP and monitored regarding survival; $*p < 0.01$ compared to the mice with a transplant of *CD300f*$^{-/-}$ BMMCs. (**b**) Numbers of neutrophils recruited into the peritoneal cavity (n = 4 per group). The data are expressed as mean ± SD; $*p < 0.01$ (Student's *t*-test). The data are representative of two independent experiments. (**c**) Numbers of mast cells among peritoneal lavage cells from *Kit*$^{W-sh/W-sh}$*CD300f*$^{-/-}$ mice with a transplant of WT or *CD300f*$^{-/-}$ BMMCs (each, n = 6). The data are representative of two independent experiments. The data are expressed as mean ± SD.

Figure S9. Transfusion of WT or *CD300f*$^{-/-}$ BM-derived macrophages equally improved the survival of macrophage-depleted WT mice after CLP. Macrophage-depleted WT mice were intravenously injected with 10^6 WT or *CD300f*$^{-/-}$ or PBS as a control (n = 7 per group) 6 h before CLP induction and were monitored regarding survival. $*p < 0.01$ compared to CLP-operated control mice; n.s.: not significant (long-rank test).

Supplementary Fig. S1

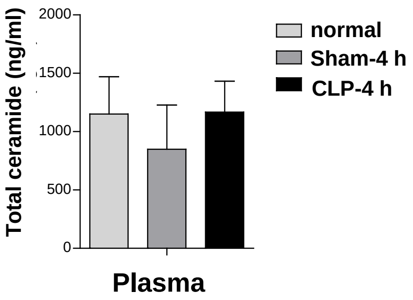

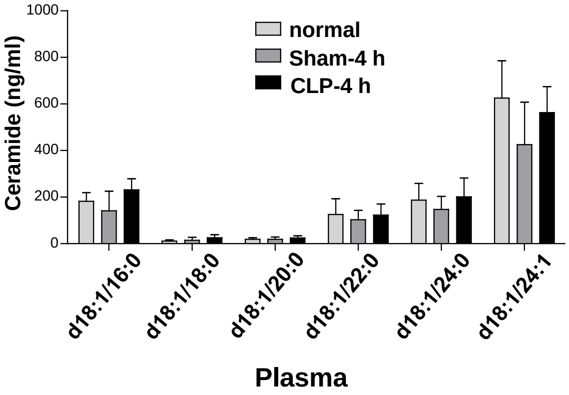

Supplementary Fig. S2

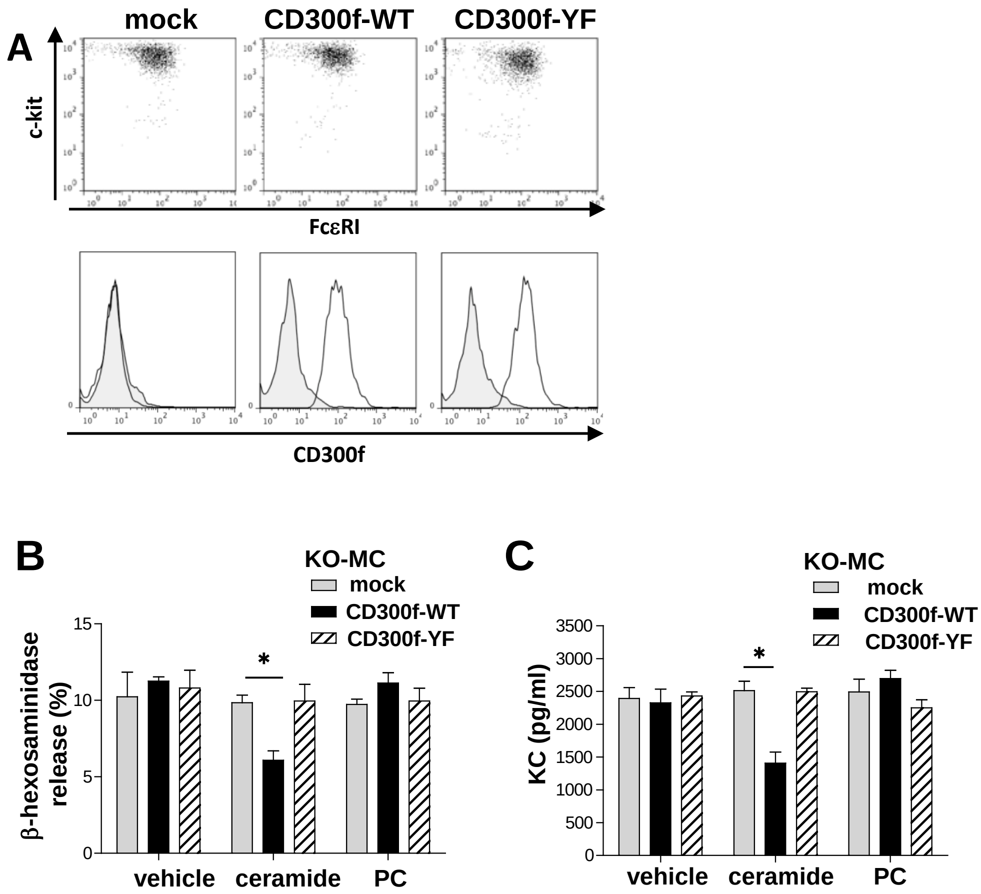

Supplementary Fig. S3

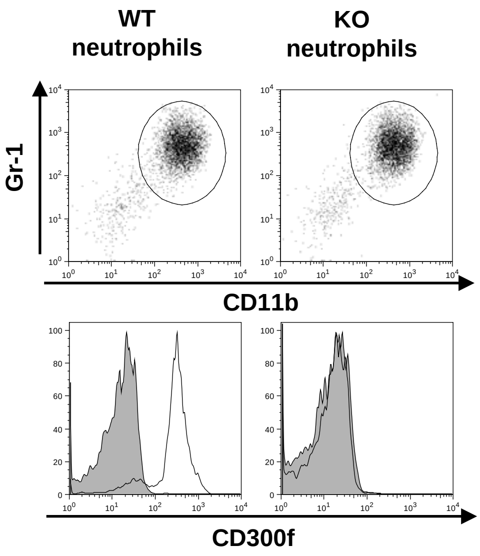

Supplementary Fig. S4

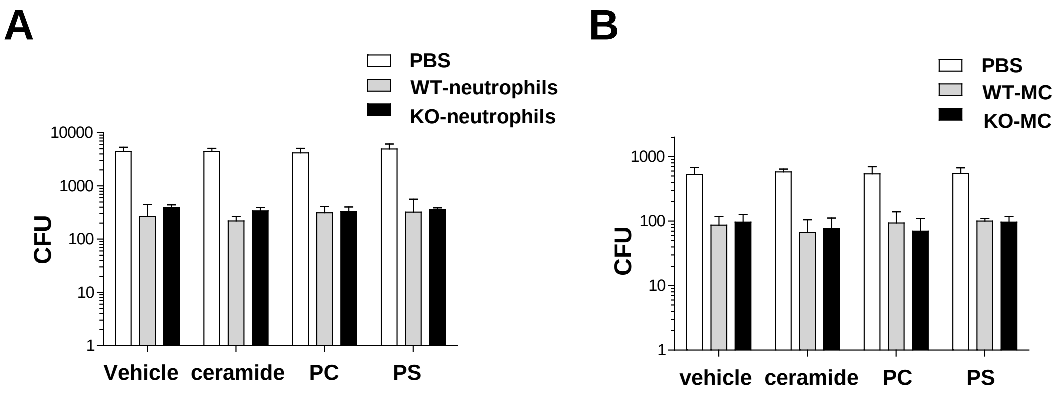

Supplementary Fig. S5

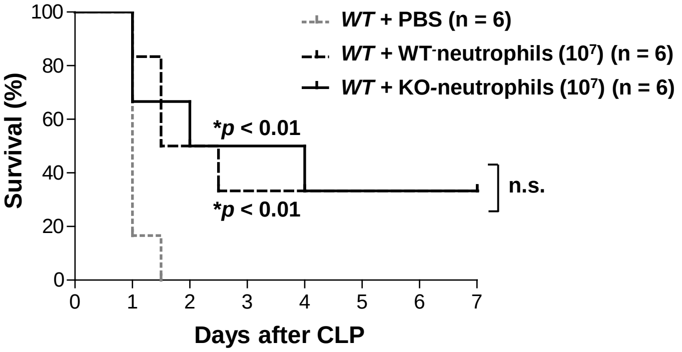

Supplementary Fig. S6

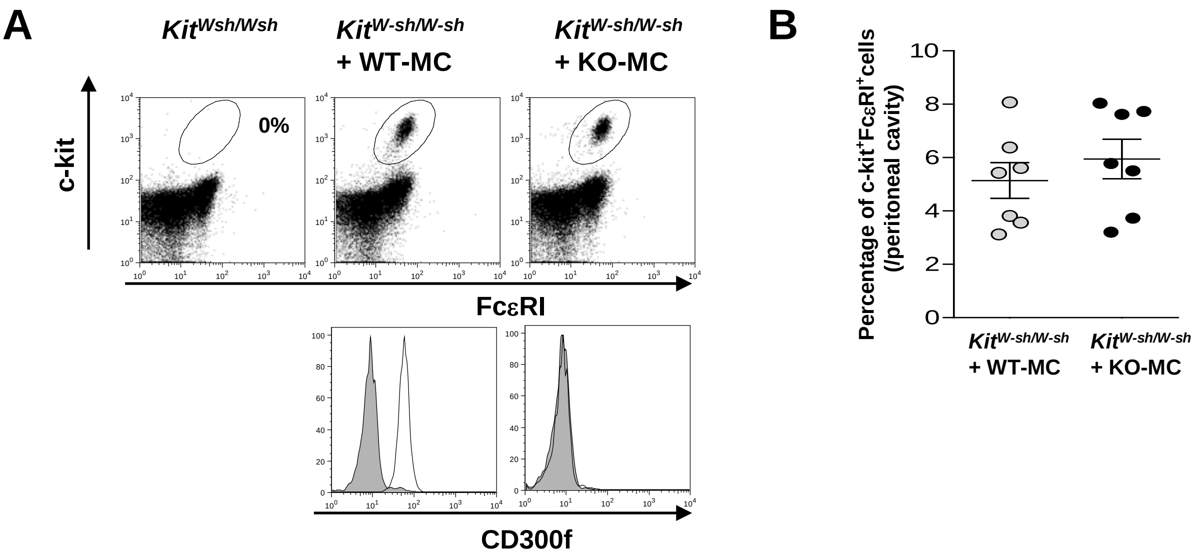

Supplementary Fig. S7

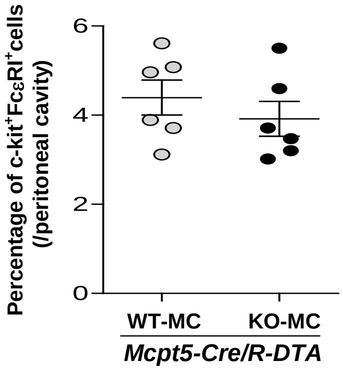

Supplementary Fig. S8

A

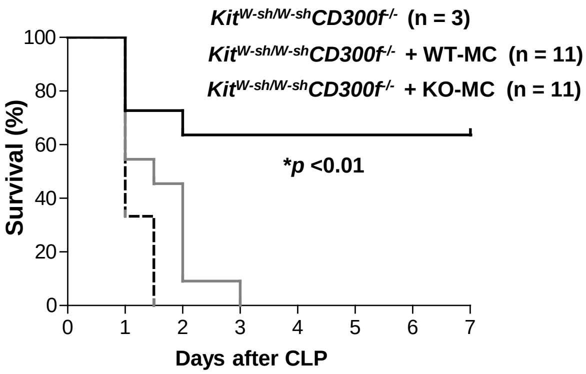

B

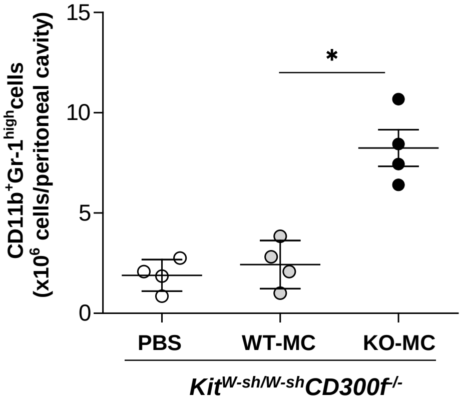

C

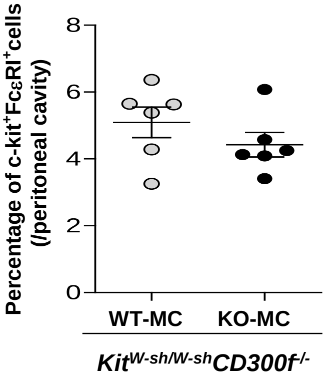

Supplementary Fig. S9

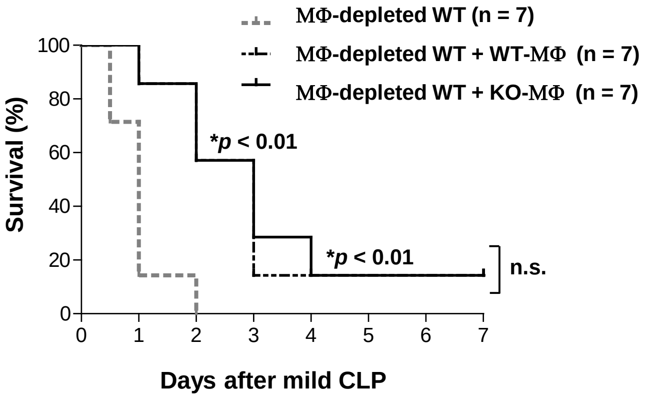

Supplement: Supplementary file 1 — Supplemntal figures and figure legends [file 41598_2017_4647_MOESM1_ESM.pdf]
